# Microscale walkability indicators for fifty-nine European central urban areas: An open-access tabular dataset and a geospatial web-based platform

**DOI:** 10.1016/j.dib.2021.107048

**Published:** 2021-04-21

**Authors:** Alexandros Bartzokas-Tsiompras, Yorgos N. Photis, Pavlos Tsagkis, George Panagiotopoulos

**Affiliations:** a National Technical University of Athens, Zografou Campus, 9 Iroon Polytechniou str, Zografou, 15780, Greece; b Metsovio Interdisciplinary Research Center, National Technical University of Athens, Zografou Campus, 9 Iroon Polytechniou str, Zografou, 15780, Greece

**Keywords:** Urban Indicators, Walkability, Physical activity, City center, European Cities, Urban Mobility, Pedestrian Planning, Urban Design

## Abstract

A growing body of empirical findings suggests that more satisfactory, compact, and traversable built environments can positively influence active travel, physical activity, and the walking experience. To this end, planning for better and more walkable places has been identified as a hot topic in urban studies and public health research, since. However, European-level indicators assessing aspects of pedestrian-friendly urban environments are largely lacking. This article introduces spatial and tabular data files of 17 pre-processed and microscale walkability indicators. The dataset presents relevant to the pedestrian environment information for 59 central urban areas from 26 European countries and aims to support policy analysis and assessment related to healthy and low-carbon transportation systems as well as sustainable communities. Methodologically, we applied a virtual (i.e., Google Street View) street audit tool, block-by-block and on both sides of each street and crossing segment separately. To this end, we digitized in polyline features observations and evaluations for a total of 112.577 street- and/or crossing-segments. The data collection process was a demanding and challenging process, which lasted for 21 months and involved 46 trained observers. The data tables in this paper present processed data of each audited item topic as a total share of street segments or crossings length by city. More specifically, the data tables contain indicators that describe the following seventeen themes: percent of segments with predominant commercial or/and entertainment buildings (active uses), percent of segments with access to park/plaza, percent of segments with transit stop(s), percent of segments with available public seats, percent of segments according to their street lighting conditions, percent of segments with well-maintained buildings, percent of segments where graffiti is not present, percent of segments where a bike lane is present, percent of segments where a sidewalk is present, percent of segments with well-maintained sidewalks, percent of segments with sidewalk buffers, percent of segments according to shading levels, percent of segments with wider sidewalks, percent of segments according to the number of road traffic lanes, percent of crossings with a pedestrian walk signal, percent of crossings with curb(s) ramp and percent of crossings with a marked pedestrian crosswalk. Additionally, a dedicated web-GIS platform has been designed and developed to visualize and disseminate collected data in openly available density maps of high spatial resolution (50 m × 50 m). The above data can be utilized to both raise awareness of unsatisfactory pedestrian environments and appoint them as a key health and environmental issue, as well as to assist European policy-makers to apply urban mobility strategies and monitor progress in urban sustainability and public health goals.

## Specifications Table

**Table:** 

Subject	Planning and Development
Specific subject area	Microscale walkability audit tools: An in-field or online data collection method, also known as street observation instruments, to assess microscale environmental factors that play an important role in increased physical activity and active travel [1]. Microscale walkability data enable the detection of unhealthy and unsatisfactory pedestrian environments [2], [3]. Thus, these tools are considered as suitable performance indicators when planning and promoting public health and transport strategies [1].
Type of data	Aggregated tables & aggregated geospatial vector files
How data were acquired	We used a brief and validated street observation instrument [2], [3], [4] to assess 17 micro-level walkability indicators for 59 European central urban areas. The complete audit item list is presented in Table A-1 in the Appendix. In order to conduct the street audits we trained 46 observers and utilised the free online geospatial service of Google Street View (GSV). The collected block-by-block street ratings were digitized in polyline vectors and stored in a GIS database. Finally, segment-level GIS vector files were further processed and analysed in order to formulate the above mentioned walkability indicators.
Data format	Spreadsheet: Processed/aggregated data tables in .xls format Geospatial: Processed/aggregated geospatial data in .geojson format
Parameters for data collection	The raw GIS data were collected from 59 central urban areas across Europe. Unfortunately, in this research we were not able to consider cities from the following EU countries: Germany, Finland, Malta, and Cyprus since either their GSV service was significantly outdated or the GSV image coverage of their respective streets was deficient. The boundaries of the most central administrative districts of the above cities delineated our study areas (e.g., Madrid Centro, Oslo Centrum, Brussels Pentagon District, etc.). For cities where such a boundary was absent or difficult to be identified, it was represented by a 15 min walking distance service area polygon originating from the most popular and centrally located landmark of the city (e.g., Palatul Parlamentului in Bucharest). In a similar way, for cases where GSV image data were missing or problematic (e.g., objects totally blocking the view of the sidewalk, blurred images, etc.) the corresponding footpaths were not rated.
Description of data collection	Street-level data were collected between January 2019 and September 2020. The presented aggregated - secondary data indicators were pre-processed in October 2020. In practice, we adopted the brief version of the Microscale Audit of Pedestrian Streetscapes (MAPS-mini) [2] to which we added two extra items. Namely, sidewalk width and number of traffic lanes. We opted to use the specific audit tool since it is both a validated instrument [2], [3], [4], [5] as well as commonly used in physical activity and walkability studies. In essence, it is considered as a brief but robust tool suitable for massive data collection. The audit list included seventeen items related to micro-level characteristics of neighbourhood design, such as sidewalks, crosswalks, lights, curb cuts, street furniture, parks, transit, aesthetics. Observers completed each item-list by block-by-block recording their virtual ratings in a GIS database. Each recorded street observation was separately pertained to both sides of all streets and crossing segments in the district. The application of the audit tool was conducted online, using the GSV service. On this basis, 46 observers (i.e., undergraduate and postgraduate students) were trained on how to implement the specific method. To ensure homogenous and robust ratings among the cities and the auditors, we also employed and trained a supervising team of four experienced researchers (i.e., PhD students or planning/architecture professionals). In essence, supervisors played the role of trainers, data quality auditors, and advisors accordingly and with respect to the observers. To this end, the supervising team validated data collection samples per each auditor periodically, proposed possible data quality improvements, and verified the auditors ability to follow the instructions and rules of the data collection process effectively.
Data source location	The presented data refer to 59 central urban areas from the following cities: Amsterdam, Antwerp, Athens, Barcelona, Bari, Bilbao, Birmingham, Bologna, Bordeaux, Bratislava, Brno, Brussels, Bucharest, Budapest, Copenhagen, Dublin, Florence, Glasgow, Gothenburg, Krakow, Leeds, Lille, Lisbon, Ljubljana, London, Luxembourg, Lyon, Madrid, Malaga, Malmo, Manchester, Marseille, Milan, Montpellier, Nice, Oslo, Palermo, Paris, Plovdiv, Poznan, Prague, Riga, Rome, Rotterdam, Seville, Sofia, Stockholm, Strasbourg, Tallinn, Thessaloniki, Toulouse, Turin, Valencia, Vienna, Vilnius, Warsaw, Wroclaw, Zagreb, Zurich. The geographical boundaries for each analyzed central urban area can be viewed online by following the link: http://geochoros.survey.ntua.gr/walkandthecitycenter/app-map
Data accessibility	1) Tabular dataset (CC BY 4.0 licence): Repository name: Mendeley Data Direct URL to data: http://dx.doi.org/10.17632/pvtwcjs365.2 2) Geospatial dataset (CC BY-NC 3.0 AU license): Repository name: Mendeley Data Direct URL to data: http://dx.doi.org/10.17632/prztv3jb2v.1 The aggregated geospatial data as well as the boundaries of each study area are also available for preview, share, and download in either .shp or .geojson format through a dedicated WebGIS platform, hosted on a National Technical University of Athens server. URL link: http://geochoros.survey.ntua.gr/walkandthecitycenter/home

## Value of the Data

• Although there is a significant policy interest for healthier, more sustainable, inclusive, and resilient transportation systems [6], [7], [8], there is a significant data scarcity in comparable and street-level walkability indicators [9]. The lack of relevant indicators across Europe and places with heterogeneous characteristics constitutes an important barrier in the generalizability of walkability research findings [9,10]. On this basis and to our knowledge for the first time, our dataset enables micro-level walkability assessments and comparisons among a diverse group of European central urban areas and cities. Furthermore, it can assist urban areas facing significant pressures on their mobility systems to plan more appropriate strategies and deliver more walking-friendly and sustainable environments by benefitting from such data-driven frameworks.
• Decision makers, stakeholders and urban policy analysts from the following sectors can significantly benefit from the provided datasets: researchers, urban and transport planners, public health practitioners, decision makers and city councils, NGOs, think tanks, journalists.
• The reusability of the dataset can support the construction of composite indicators and city rankings [11] related to urban sustainability issues. It also encourages cross-city and peer-city comparisons in pedestrian planning issues across Europe.
• The entire geospatial dataset can additionally be utilized by local planners across Europe in order to identify hot/cold spots of low/high walkability patterns and accordingly propose cost-effective and more realistic solutions and strategies.
• By detecting significant disparities in their pedestrian environments these data can substantially support cohesion policies targeting inclusive, healthy, and sustainable urban development for European cities.

## 1 Data Description

The data in this paper have been pre-processed and aggregated and are provided in two formats. Namely, as tables in a spreadsheet (.xlsx) and as geospatial data in .shp or .geojson.

**Fig. 1:**
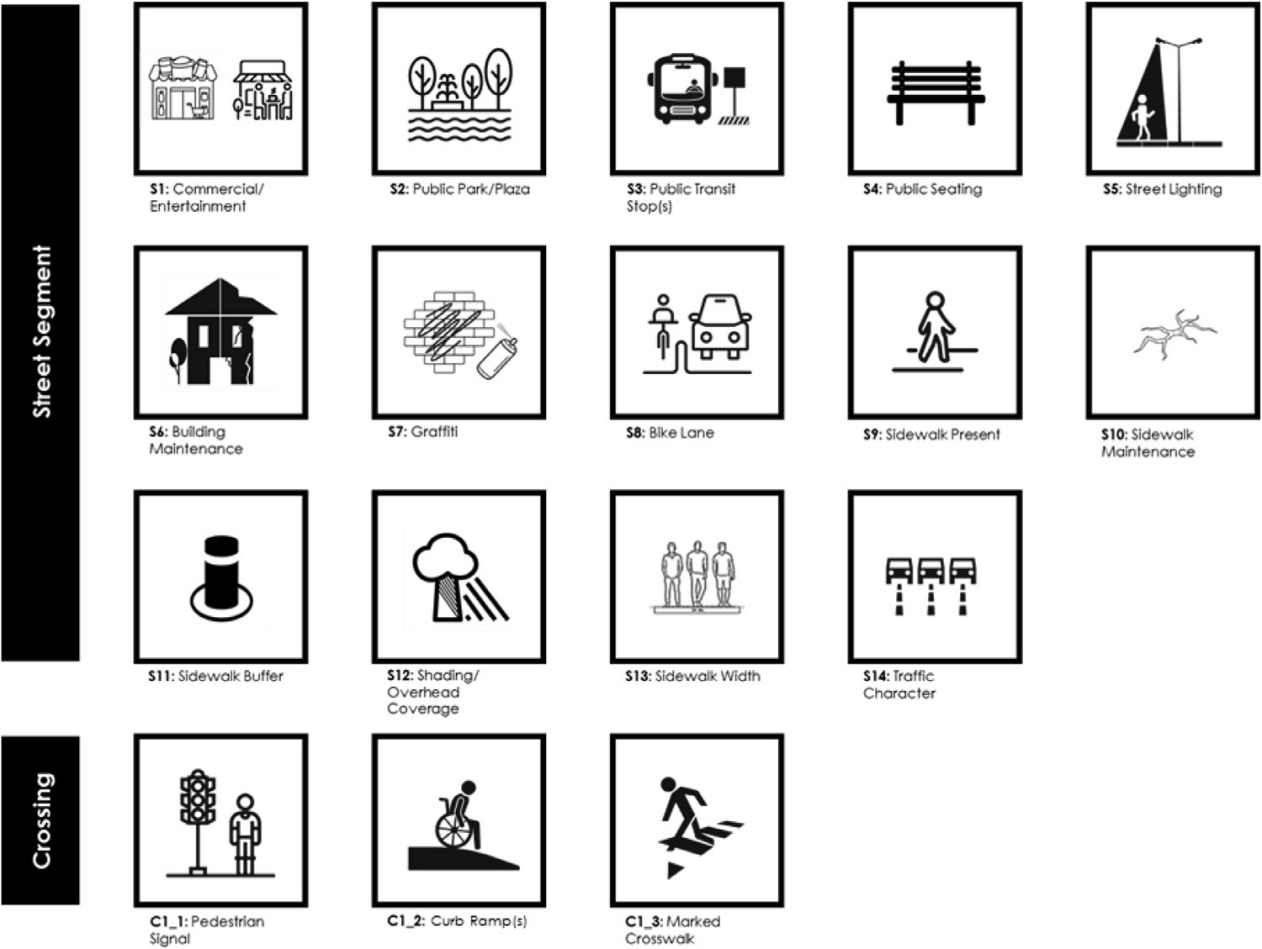
Visualization of the topics covered by the data indicators.

Table 1 summarizes some fundamental characteristics of the conducted research during which and by utilizing Google Street View (GSV) imagery data we analyzed 59 central urban areas, from 26 European countries, audited 112.577 segments with a total length of 9.822 km, trained 46 observers, employed 4 Supervisors. The duration of the primary GIS data collection phase was 21 months (January 2019 – September 2020). The resulting aggregated data indicators can be useful to both planners and decision makers interested in micro-level walkability assessments. All indicators are related to policy-amenable aspects of the microscale pedestrian environment and ultimately aim to support the design of more healthy and sustainable cities. The topics of the described datasets are briefly illustrated in Fig. 1.

**Table 1:** Summary characteristics of the research project.

Total number of analyzed central urban areas	59
Total number of involved European countries	26
Total number of audited segments	112.577
Total length of audited segments (in km)	9.822
Online source of street images	Google Street View (GSV)
Total number of employed supervisors	4
Total number of trained street observers	46
Duration of the street observation process (in months)	21

**Fig. B-1:**
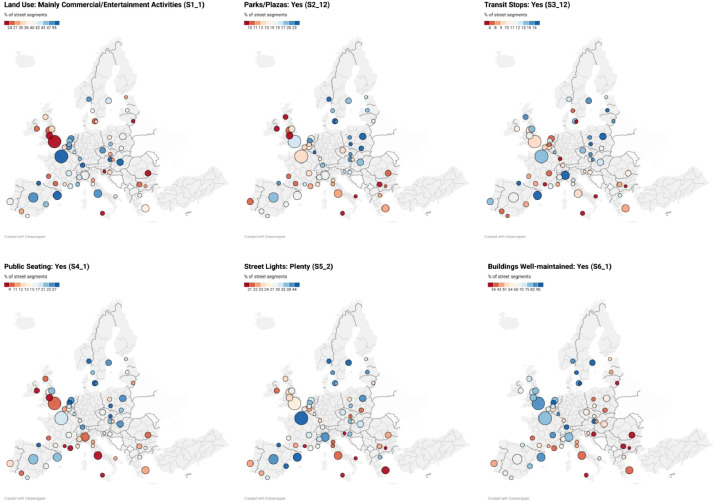
European-level quantile (*n* = 10) maps of indicators S1_1, S2_12, S3_12, S4_1, S5_2, S6_1 (indicator codes are available in the supplementary tabular dataset).

**Fig. B-2:**
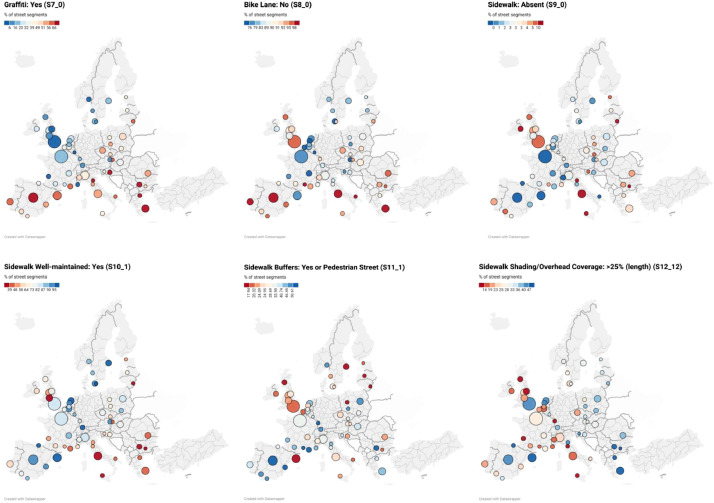
European-level quantile (*n* = 10) maps of indicators S7_0, S8_0, S9_0, S10_1, S11_1, S12_12 (indicator codes are available in the supplementary tabular dataset).

**Fig. B-3:**
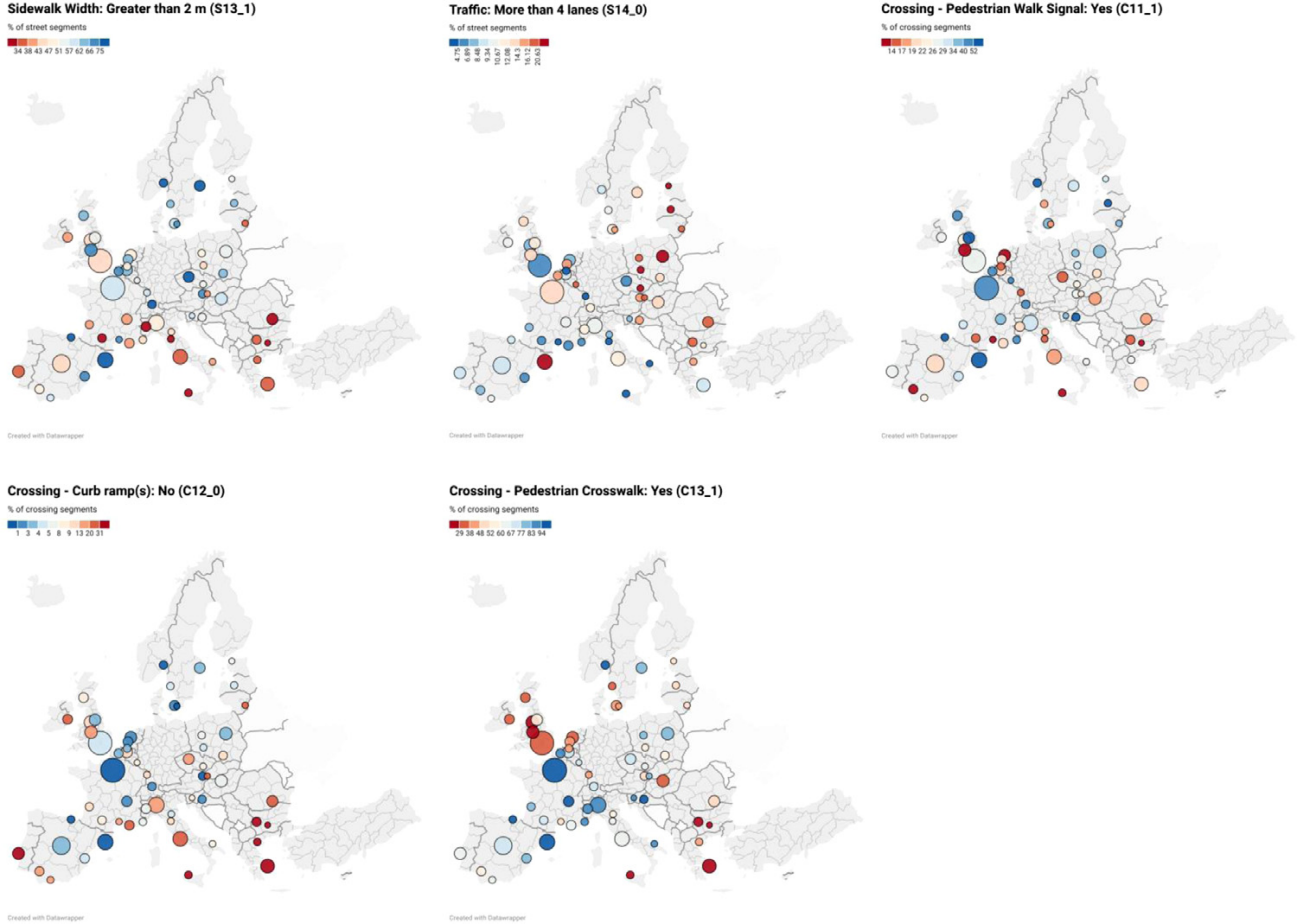
European-level quantile (*n* = 10) maps of indicators S13_1, S14_0, C11_1, C12_0, C13_1 (indicator codes are available in the supplementary tabular dataset).

In the Microsoft Excel Workbook (.xlsx) ‘WALKABILITY_DATA_BARTZOKAS-TSIOMPRAS_et.al_2021.xlsx’ the worksheet ‘total_descriptive_statistics’ presents some basic information by city with regard to the audited area size, the total number of audited street- and crossing-segments and the no data records of the primary GIS database. In the ‘GSV_stats’ worksheet rows demonstrate the share of audited segments in each district while columns represent the year of GSV image used. The ‘data_indicators’ worksheet contains the summarized results of the auditing process per central urban area and per item included in the audit tool's list. All data values in this table are expressed in weighted total shares of each central urban area's streets or crossings segments and all indicator columns refer to specific codes in brackets providing data reference information. In addition, European-level quantile (*n* = 10) maps of selected indicators can be seen in Fig. B-1, B-2, B-3 in the appendix.

Furthermore, we provide open access (CC BY-NC 3.0 license) to pre-processed geospatial data grids of high spatial resolution (50 m X 50 m) through our Walk and the City Center WebGIS platform. Τhe above mentioned geospatial data include both the micro-level walkability indicators (see Fig. 1) and the boundary polygons of each analyzed central urban area, while users can download either each selected indicator map per city (.geojson) or the entire dataset including all^1^ cities and indicators (.shp). A detailed description of the contents of its attribute table fields is provided in the accompanied read_me.txt file.

**Fig. 2:**
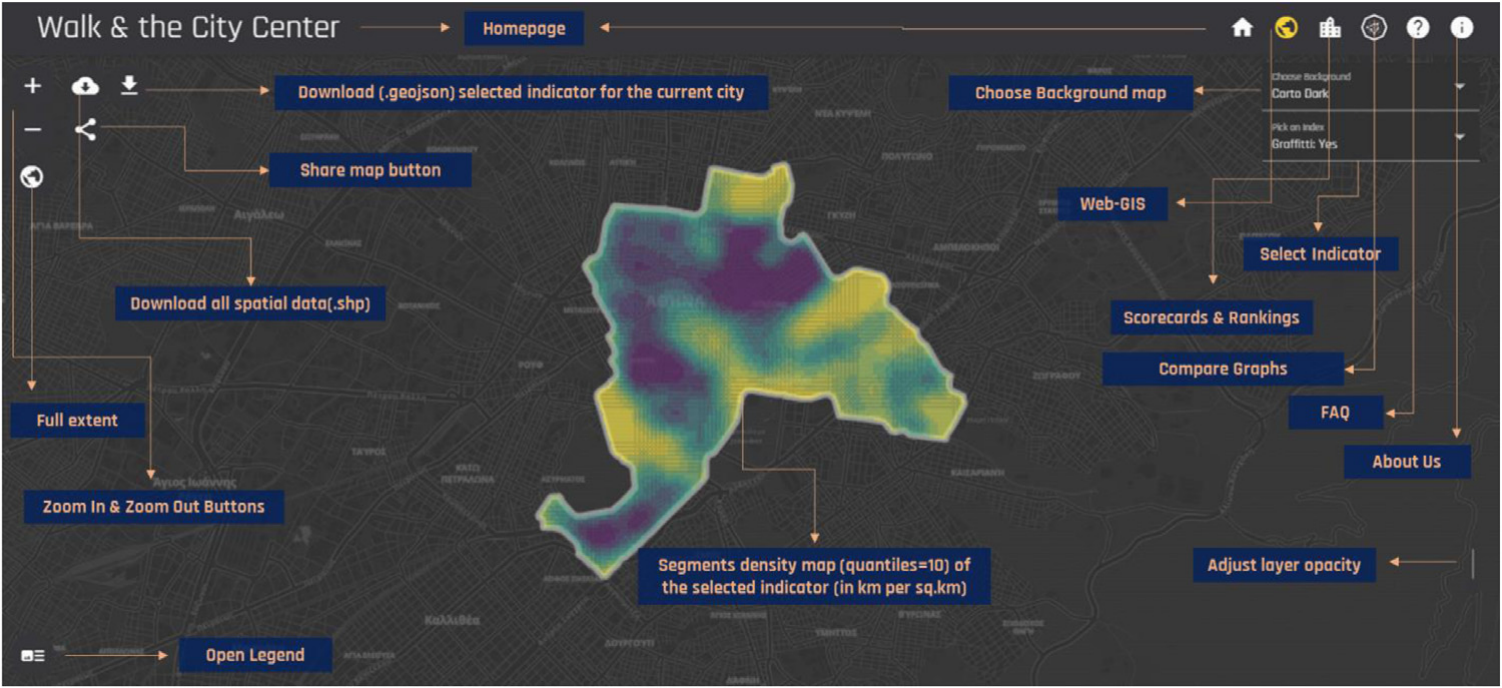
Preview of the geospatial platform and the provided spatial dataset in density (decile) maps (URL link: http://geochoros.survey.ntua.gr/walkandthecitycenter/app-map).

**Fig. 3:**
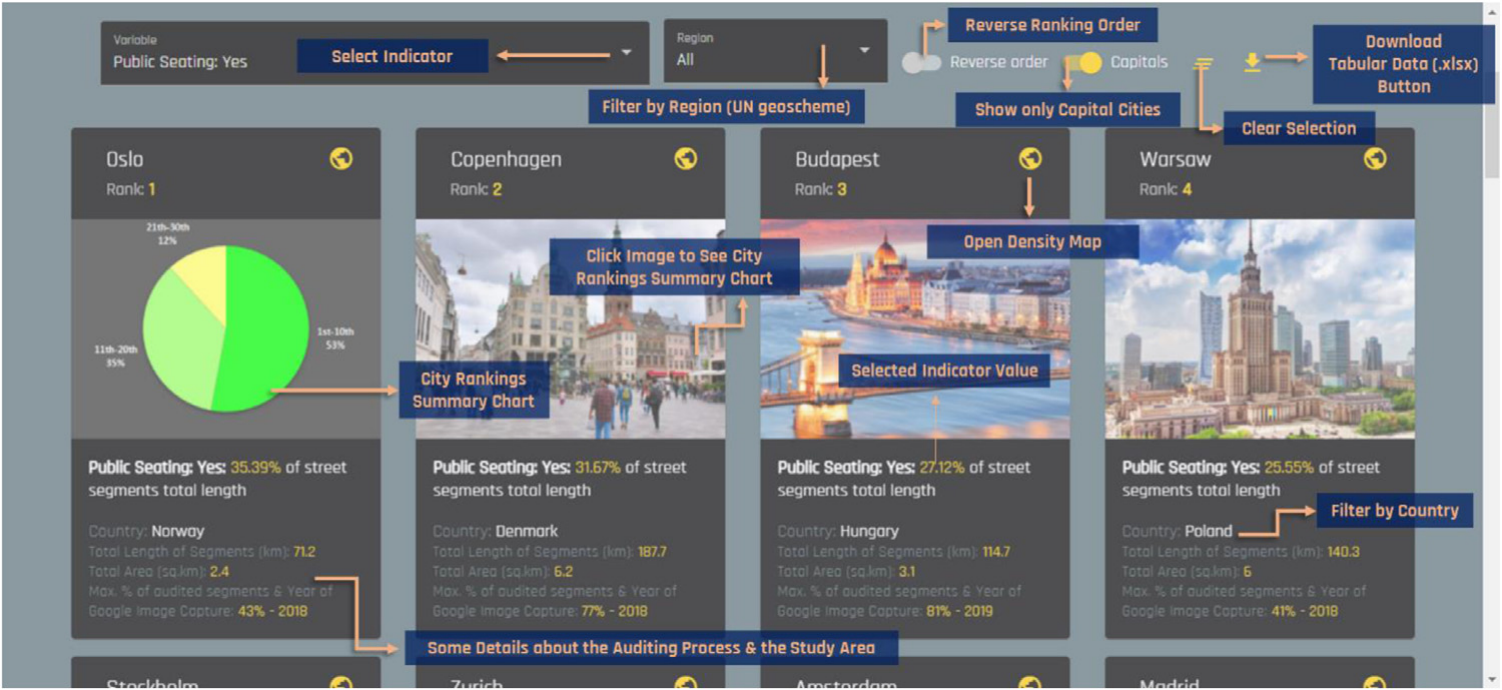
Preview of the web-platform and the ranking function of cities per each indicator (URL link: http://geochoros.survey.ntua.gr/walkandthecitycenter/cities).

**Fig. 4:**
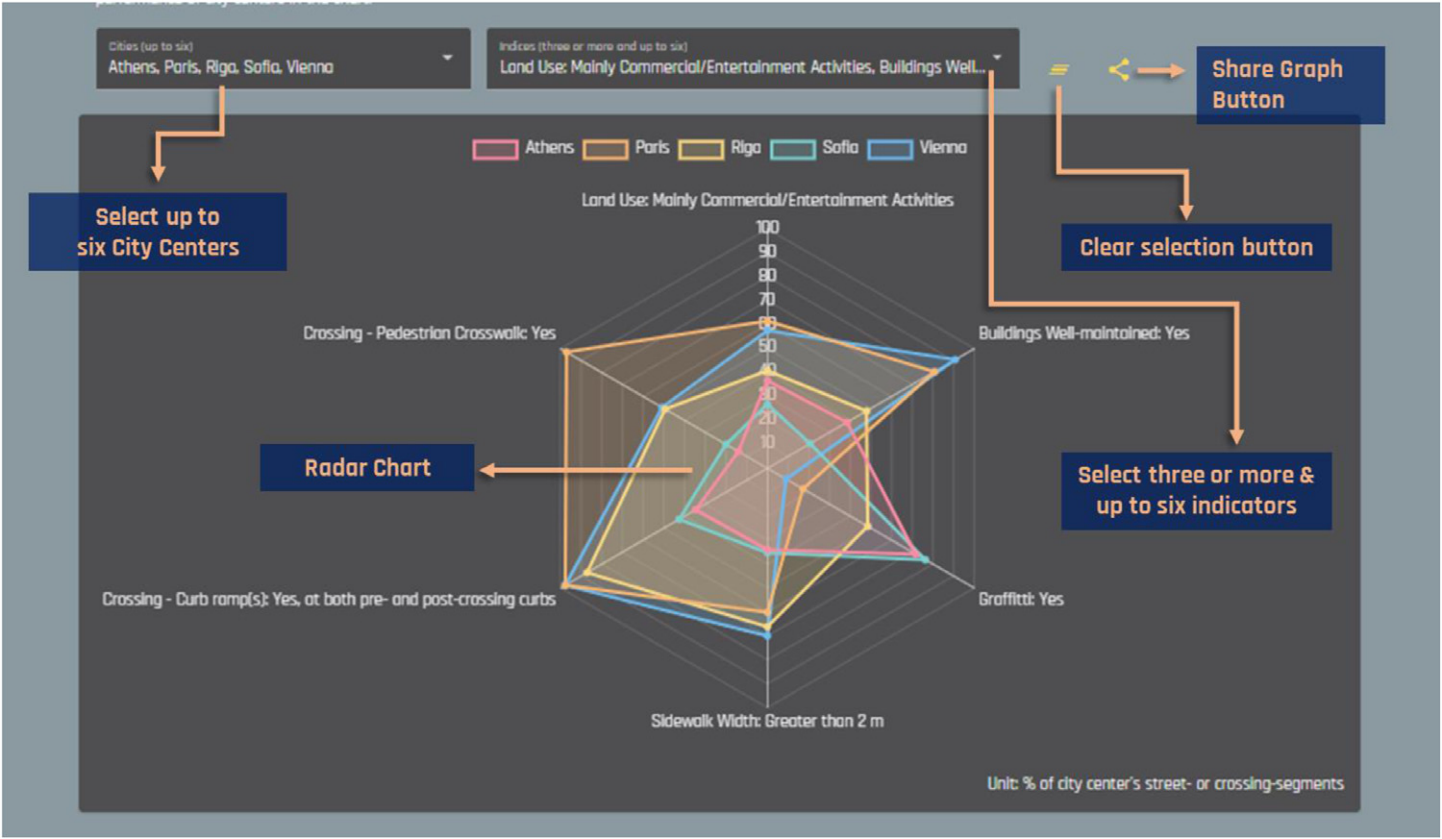
Preview of the web-platform and the compare graphs tool (URL link: http://geochoros.survey.ntua.gr/walkandthecitycenter/chart).

More specifically, the geospatial grids have been calculated by applying the Kernel density tool (search radius: 250 m.) in ArcGIS Desktop, v.10.3 (ESRI, REDLANDS) to the raw GIS vectors (i.e., polylines), created during the street observation phase of our research. We decided to implement the specific spatial analysis process in order to smoothen the collected information in a way that will be easier to visualize and interpret. A snapshot of the geospatial online map is illustrated in Fig. 2. Additionally, users of the web-based platform can also rank cities according to their performance in each indicator included in the tabular dataset (see Fig. 3) as well as compare cities performance with respect to up to six of the indicators (see Fig. 4).

## 2 Experimental Design, Materials and Methods

### 2.1 Micro-level walkability audit tool method

A walkability audit tool method was employed to collect the raw GIS data described in this article. In general, these tools are observational instruments and they are used to assess quantitatively and qualitatively deficiencies in the pedestrian environment and infrastructure [12]. Such an approach provides detailed data on microscale environmental factors that seemingly increase walking and physical activity behaviours [5]. Audit tools are popular in community planning practice, since they examine easily modifiable factors of the built environment and they do not require to transform macro-level walkability features such as neighbourhood layout or land-use and population spatial patterns [9,10,13]. Examples of more analytical reviews regarding micro-level walkability audits can be found in the work of Brownson et al. [1] and Aghaabbasi et al. [12].

**Fig. 5:**
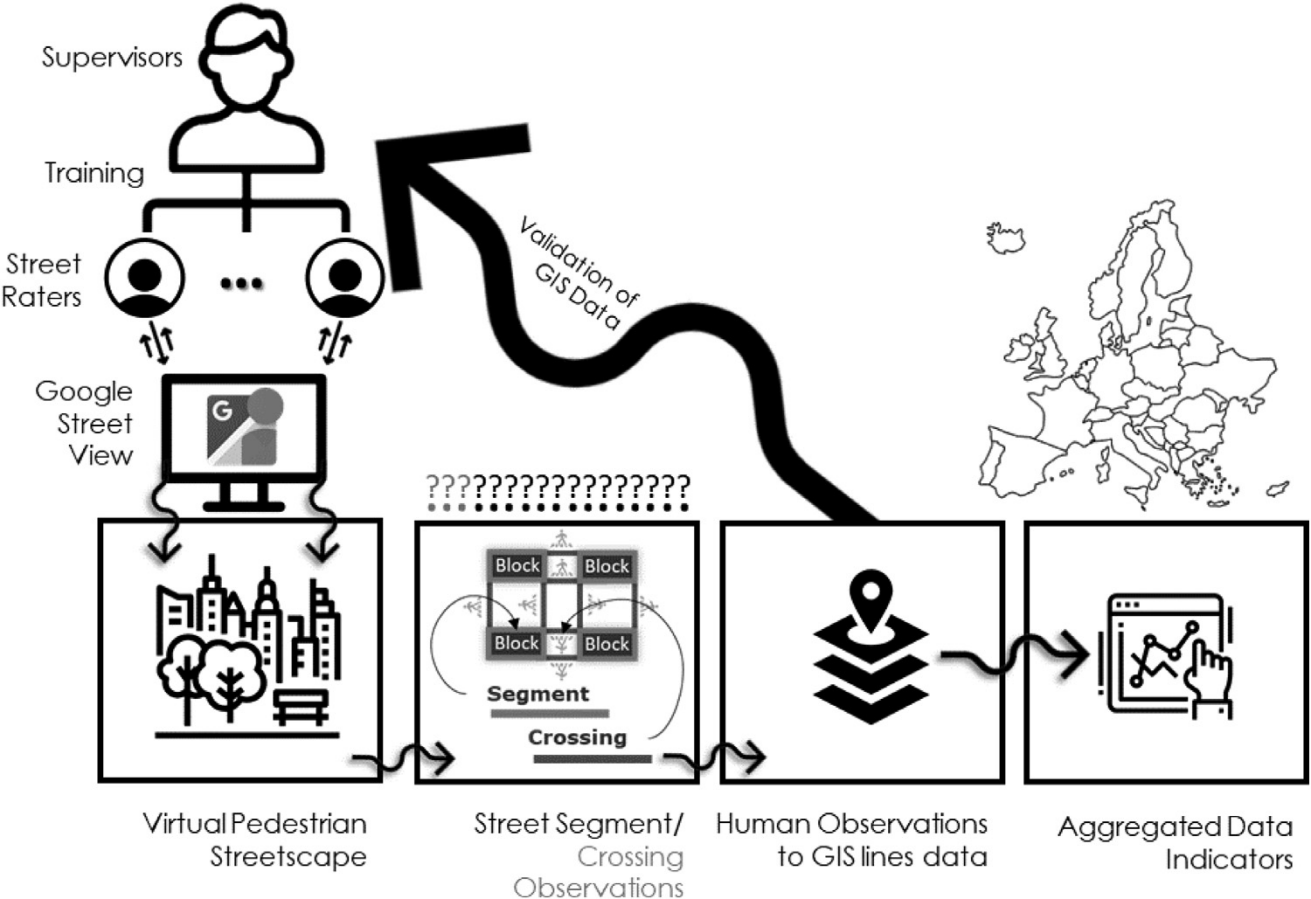
Data collection process.

In essence, we opted to apply the brief version of the Microscale Audit of Pedestrian Streetscapes (MAPS-mini) [2] for three reasons. Firstly, it is quick and suitable for massive data collections [3] secondly, it has been previously validated as an acceptable method for physical activity and walkability studies [2,3,5] and thirdly, it is a reliable method for virtual assessments by auditors who are not familiar with the specific study area [3,4]. For the purpose of our research and in order to match our approach, certain items in the original MAPS-mini list have been slightly modified while we have also added two extra features related to the sidewalk's width and the road's traffic levels. The complete audit items list deployed in this research is described in Table A-1 in the Appendix A where the first 14 items are related to the street segment part of each block side and the last 3 items pertain to questions regarding the crossing segments (parts of street intersections) (see also Fig. 5).

The graph in Fig. 5 illustrates the methodological steps followed to conduct the data collection process. According to it, we firstly recruited 4 experienced researchers (i.e., PhD students and planning/architecture professionals) as Supervisors. Supervisors train and assist their teams as well as assess and validate the data submitted by each of their auditors. Followingly, we recruited 46 observers (i.e., undergraduate and post-graduate students) and trained them during a two day workshop seminar in order to ensure that the rules and the practicalities of the walkability audit process have been clearly explained and well understood. However, since the recruitment of the auditors was not an one step process, we actually had to recruit and train smaller teams of observers eight times during the project. Each and every participant of the auditing process was consistently and periodically assessed as well as supported by their assigned supervisor. The observers had to timely submit their data to their supervisor for quality assessment purposes while supervisors had to provide feedback reports for possible data improvements and corrections. In case of low quality datasets^2^ the specific area's audit data collection process was restarted from scratch and inevitably assigned to a different observer. During this phase, a total of seventeen low quality datasets were submitted from initially recruited observers which were finally excluded. It should be mentioned that in each district we deployed varying numbers of auditors (1 up to 4) relative to its size and estimated data volume. Last but not least, all auditors were asked to measure individually the average time spent to complete the audit tool list for one street and crossing segment. Since the auditing time with the proposed method significantly relied on the experience of each auditor, they measured and reported their average auditing time only when the 75% of the area assigned to them was completed. To this end, we calculated the average time required by the 46 observers to complete the audit list in one street and crossing segment which was 1 min and 39 s. However, at the end of the project the majority of team members admitted that during the first days of applying the audit tool they actually needed more than triple this average time in order to evaluate each street and crossing segment.

At the beginning of the auditing process every observer was supplied with his/her assigned area's GIS shapefiles (ESRI) namely, the street centerlines data and transit stops locations from openstreetmap.org and a polygon shapefile with building-blocks layouts [14]. Then, each auditor was asked fisrt, to systematically observe the streetscape and second, to record his/her evaluations/answers to 17 simple questions (audit items, see Appendix Table A-1) in a GIS database . In essence, the questions of the audit required each auditor to indicate whether a specific characteristic was present or not, to count the frequency of an amenity or to assess the maintenance conditions of an infrastructure. In other words, observers were asked to virtually assess the pedestrian environment features block-by-block and followingly to digitize their answers/evaluations for both sides of each segment of the street, as separate polyline GIS data (ArcGIS for Desktop, v.10.3, ESRI, Redlands, CA).

**Fig. 6:**
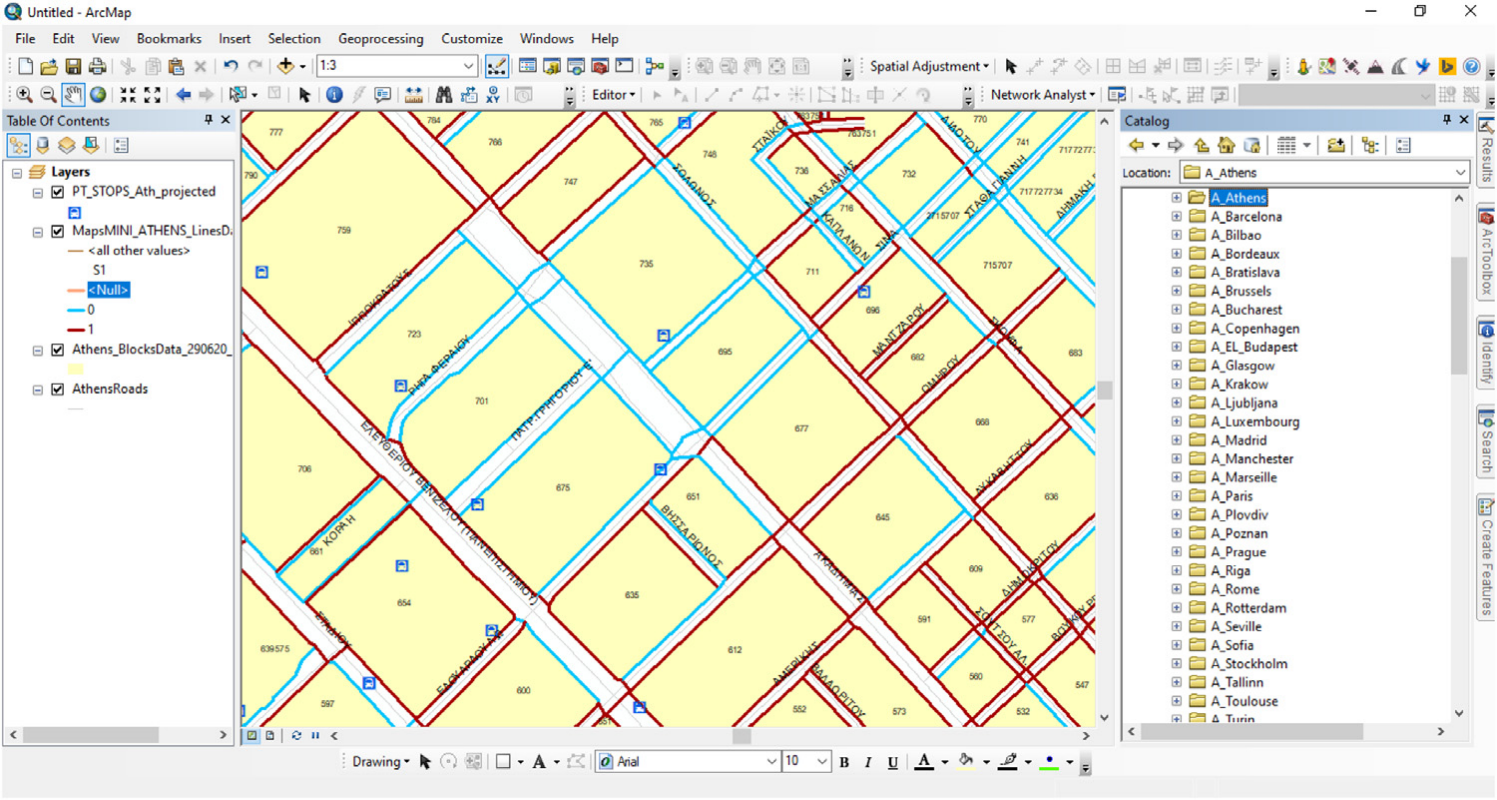
A screenshot from the GIS environment presenting the structure of the street- and crossing-segments (raw) GIS data.

**Fig. 7:**
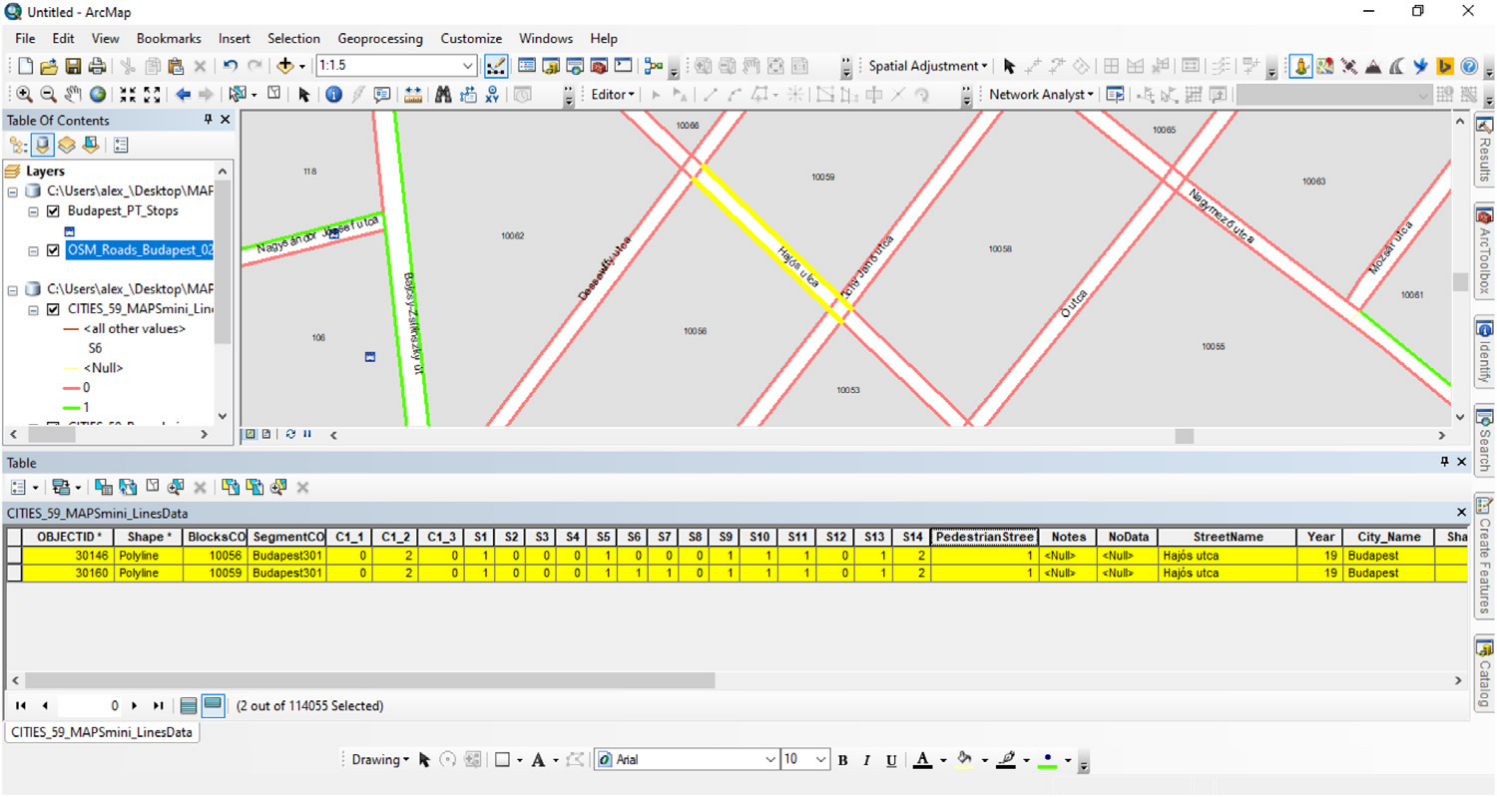
A screenshot from the GIS environment that demonstrates the structure of the attribute table.

Therefore, the created polyline shapefile had an attribute table containing 17 fields with the above descriptive characteristics. Geographic features in the shapefile (polylines) correspond to either a street or crossing segment of a central urban area (see Figs. 6 & 7), while the tabular file incorporates the street observation evaluations (see Fig. 7). In particular, Fig. 6 shows a screenshot image from the GIS environment during the auditing process and demonstrates the structure of the layers and the created geographic features while Fig. 7 illustrates two records from the tabular dataset. A tutorial video of a typical virtual street assessment example can be viewed in the following URL: https://youtu.be/VqIb1hJwerU?t=4.

Finally, further processing of the raw GIS polyline features allowed us to calculate the aggregated indicators included in the data tables of this article. To this end and in order to calculate aggregated measures and values we applied the following formula:

$$I_{y}=\sum_{a=1}^{n}\frac{x_{a}}{b}$$

Where

I is an audit item (indicator) and y is its evluation
a is a segment evaluated with y for audit item I
n is the last segment evaluated with y for audit item I
x is the segment length
b is the total length of audited segments in the area

### 2.2 Programming concept of the geospatial data platform

The geospatial platform of this project was designed and developed in order to disseminate as well as visualize both the tabular and raster datasets (50 m x 50 m), and its source code is hosted on Github (https://github.com/geochoros-ntua/WalkAndTheCityCenter). Our programming concept was based on the client-server architecture as three-tier architecture. Client-server three-tier architecture is made up of the application server (Application Tier), database server (Data Tier) and PC (Presentation Tier). In all phases of the platform development we have followed the Object Oriented Programming paradigm (OOP) which refers to the software design and coding model of computer programming according to which software developers define objects as data types and structures, as well as routines that can be applied to these objects. All individual subsystems operate independently. In this way we achieve maximum flexibility and modularity. We have used common and widely used protocols either for the communication between the platform's internal subsystems or for communication with third party services like Open Street Map (OSM), Google Maps and Nominatim Geocoder (OGC, wms, wfs, xml, http). Finally, various technologies and third party projects have been used to develop the platform and accomplish its goals and objectives. PHP programming language has been utilized on the server side and JavaScript for the client side. Most of the third party libraries used are projects running on the client and a few on the server side. All of the third party modules and frameworks are Open Source projects (OS) and their selection was based on its contributor's investment, usage wideness, documentation as well as on its development activity. The third party OS libraries used in this platform are: Openlayers for the WebGIS implementation and capabilities, Geostats.js for the geographical statistical classification, Ol-ext to extend openlayers's functionality, Open Street Map (OSM) and Google Maps in order to use various base maps within the web-GIS toolkit, Jstat.js for the tabular statistical analysis and finally, Angular and Node.js for the development.

## Supplementary Materials

Supplementary material associated with this article can be found in the online version at http://dx.doi.org/10.17632/pvtwcjs365.2 (tabular dataset) as well as at http://dx.doi.org/10.17632/prztv3jb2v.1 (spatial dataset).

## CRediT Author Statement

**Alexandros Bartzokas-Tsiompras:** Conceptualization, Methodology, Data Analysis, Data Management, Data Collection, Writing - Original draft preparation; **Yorgos N. Photis**: Conceptualization, Supervision, Reviewing and Editing; **Pavlos Tsagkis:** Writing - Original draft preparation, GIS Software, Data Analysis; **George Panagiotopoulos:** GIS Software, Data Analysis, Website Development.

## Declaration of Competing Interest

The authors declare that they have no known competing financial interests or personal relationships which have or could be perceived to have influenced the work reported in this article.

## Figures and Tables

**Table A-1:** Item list of the applied walkability audit tool.

Type of Segment	Item Code	Item Name	Item Question	Item Answers	Answer Description
Street	S1	Predominant land use type	Is this primarily a residential or commercial/entertainment segment? (Active uses)	0	More than half of the buildings in the rated segment present non-commercial and non-entertainment activities (at the ground-floor level)
				1	More than half of the buildings in the rated segment present commercial and entertainment activities (at the ground-floor level)

Street	S2	Access to public park/plaza	Is there any access to public park or/and plaza?	0	None
				1	One access point
				2	Two or more access points

Street	S3	Public transit stop(s)	How many public transit stops are present?	0	None
				1	One (when there are more than one stop of the same type of public transport, then give 1 point)
				2	Two or more stops (when there are more than two stops available, but belong to different types of public transport, then give 2 points)

Street	S4	Public seating	Is there any public seating furniture present (bus stop benches & siting places of private restaurant/cafes did not count)?	0	No
				1	Yes

Street	S5	Street Lights	What about the public street lighting conditions? (private lighting did not count)	0	Absent
				1	Normal
				2	Ample
Street	S6	Maintenance of buildings	Are buildings of the audited segment well- maintained?	0	No, at least one building is not well-maintained
				1	Yes

Street	S7	Graffiti	Is graffiti vandalism present? (murals are not counted as graffiti)	0	Yes
				1	No
Street	S8	Bike lane	Does the segment have a designated cycle lane?	0	No
				1	Yes, painted on the road
				2	Yes, the cycle lane is separated from traffic with physical barriers

Street	S9	Sidewalk	Is a sidewalk present?	0	No
				1	Yes

Street	S10	Sidewalk maintenance	Is the sidewalk well-maintained?	0	No or no sidewalk present
				1	Yes

Street	S11	Sidewalk buffer	Are sidewalk buffers present and regularly installed at least in the half length of the segment?	0	No or no sidewalk present
				1	Yes or it is a pedestrian zone

Street	S12	Tree Shading/Overhead Coverage	What proportion of the segment length is shaded by trees or other overhead coverage?	0	0–25% or no sidewalk present
				1	26–75%
				2	76–100%

Street	S13	Sidewalk Width	Is the net width of the sidewalk supportive for three individuals to walk simultaneously and in parallel?	0	No or no sidewalk present
				1	Yes

Street	S14	Traffic	What is the traffic character of the road?	0	More than 4 traffic lanes
				1	2 - 3 traffic lanes
				2	Single lane or pedestrian zone

Crossing	C1_1	Pedestrian Walk Signal	Is a pedestrian walk signal present?	0	No
				1	Yes
				Null	Check out the traffic signs and do not rate if the crossing is not interrupted by a vehicular road (e.g., pedestrian zone)

Crossing	C1_2	Curb Ramp(s)	Is there a ramp at the curb(s)?	0	No
				1	Yes, at one curb only
				2	Yes, at both pre- and post-crossings curbs
				Null	Check out the traffic signs and do not rate if the crossing is not interrupted by a vehicular road (e.g., pedestrian zone)

Crossing	C1_3	Marked Crosswalk	Is there a marked pedestrian crosswalk? (i.e., Zebra or Pelican)	0	No
				1	Yes
				Null	Check out the traffic signs and do not rate if the crossing is not interrupted by a vehicular road (e.g., pedestrian zone)

## Footnotes

1 Τhe full GIS files (.shp), containing all cities and indicators, has been uploaded as a .zip file in a trusted online repository (Mendeley Data). The .zip file also includes a read_me.txt file where users can find more information about the attribute table fields of the shapefile (metadata) and a disclaimer.txt file.

2 Item-level reliabilities of a random sample of 10% of audited segments per city were cross-assessed by a supervising team member. Thus, the threshold of 0.6 in a Cohen's kappa statistic [3,4], which is an observer agreement measure, helped us to identify low quality datasets (Kappa>0.6=Substantial item-level reliability).
